# Alpha/Beta Interferon Receptor Signaling Amplifies Early Proinflammatory Cytokine Production in the Lung during Respiratory Syncytial Virus Infection

**DOI:** 10.1128/JVI.00333-14

**Published:** 2014-06

**Authors:** Michelle Goritzka, Lydia R. Durant, Catherine Pereira, Samira Salek-Ardakani, Peter J. M. Openshaw, Cecilia Johansson

**Affiliations:** Centre for Respiratory Infections, Respiratory Infections Section, National Heart and Lung Institute, Faculty of Medicine, Imperial College London, London, United Kingdom

## Abstract

Type I interferons (IFNs) are produced early upon virus infection and signal through the alpha/beta interferon (IFN-α/β) receptor (IFNAR) to induce genes that encode proteins important for limiting viral replication and directing immune responses. To investigate the extent to which type I IFNs play a role in the local regulation of inflammation in the airways, we examined their importance in early lung responses to infection with respiratory syncytial virus (RSV). IFNAR1-deficient (IFNAR1^−/−^) mice displayed increased lung viral load and weight loss during RSV infection. As expected, expression of IFN-inducible genes was markedly reduced in the lungs of IFNAR1^−/−^ mice. Surprisingly, we found that the levels of proinflammatory cytokines and chemokines in the lungs of RSV-infected mice were also greatly reduced in the absence of IFNAR signaling. Furthermore, low levels of proinflammatory cytokines were also detected in the lungs of IFNAR1^−/−^ mice challenged with noninfectious innate immune stimuli such as selected Toll-like receptor (TLR) agonists. Finally, recombinant IFN-α was sufficient to potentiate the production of inflammatory mediators in the lungs of wild-type mice challenged with innate immune stimuli. Thus, in addition to its well-known role in antiviral resistance, type I IFN receptor signaling acts as a central driver of early proinflammatory responses in the lung. Inhibiting the effects of type I IFNs may therefore be useful in dampening inflammation in lung diseases characterized by enhanced inflammatory cytokine production.

**IMPORTANCE** The initial response to viral infection is characterized by the production of interferons (IFNs). One group of IFNs, the type I IFNs, are produced early upon virus infection and signal through the IFN-α/β receptor (IFNAR) to induce proteins important for limiting viral replication and directing immune responses. Here we examined the importance of type I IFNs in early responses to respiratory syncytial virus (RSV). Our data suggest that type I IFN production and IFNAR receptor signaling not only induce an antiviral state but also serve to amplify proinflammatory responses in the respiratory tract. We also confirm this conclusion in another model of acute inflammation induced by noninfectious stimuli. Our findings are of relevance to human disease, as RSV is a major cause of infant bronchiolitis and polymorphisms in the IFN system are known to impact disease severity.

## INTRODUCTION

Infections at mucosal surfaces need to be managed carefully by the host in order to avoid damage to barrier functions. The pathogen needs to be eradicated rapidly, but inflammation must be tightly regulated to prevent detrimental effects on organ function. Nowhere is this more evident than in the lung, where any excess cell infiltration or damage will markedly affect gas exchange. The lung is a major site of infection by viruses, and the adverse effects of dysregulated lung inflammation have a very significant impact on human health.

The initial response to viral infection in the lung and elsewhere is characterized by the production of interferons (IFNs). There are 3 types of IFNs; type I IFNs (including alpha IFN [IFN-α] and IFN-β), type II IFNs (IFN-γ), and the recently discovered type III IFNs (IFN-λ). Irrespective of type, IFNs induce cell-intrinsic antiviral responses, activate natural killer (NK) cells, macrophages, and dendritic cells (DCs), and regulate innate and adaptive immune responses (1–3). IFN-γ is mainly produced by NK and T cells, while the synthesis of type I and type III IFNs, as well as other cytokines such as interleukin-6 (IL-6) and tumor necrosis factor alpha (TNF-α), is induced in immune and nonimmune cell types upon direct recognition of viral molecules by pattern recognition receptors (PRRs) such as Toll-like receptors (TLRs) and RIG-I-like receptors (RLRs) (1–4). The effect of IFN-λ is restricted to epithelial cells at mucosal surfaces, which express the relevant receptor (1, 2). In contrast, the receptor for type I IFNs, called IFNAR, is expressed ubiquitously by all cells. IFN-α/β is produced in the lung following infection with many viruses such as Newcastle disease virus, influenza virus, respiratory syncytial virus (RSV), and human metapneumovirus (2, 5–9).

RSV is the major cause of infant bronchiolitis (10). While RSV disease manifests as a simple common cold in the majority of cases, between 2 and 3% of children develop severe bronchiolitis. The variation in disease severity seems to be mostly due to host rather than viral factors and has recently been associated with polymorphisms in several innate immunity genes, in particular many that control the IFN system (11–14). The IFN system may therefore be a key regulator of RSV-induced lung inflammation.

To test the impact of type I IFNs on viral infections, IFNAR1-deficient (IFNAR1^−/−^) mice, which lack all signaling in response to IFN-α/β (15), have been widely used. For infections such as with reovirus or Chikungunya virus, loss of IFNAR signaling is detrimental, leading to overwhelming infection and death (16, 17). For some viruses (including influenza virus and RSV), the effect of IFNAR1 deficiency is not as severe and does not impact on survival from infection (18, 19).

Whether type I IFNs are involved only in inducing an antiviral state in the lung or whether they have a more general effect in regulation of lung inflammation has not been fully elucidated. Here, we address this question by comparing inflammation in the lungs of wild-type and IFNAR1-deficient mice in response to challenge with selected TLR agonists or RSV infection. Surprisingly, in all cases IFNAR deficiency was associated with a marked decrease in the production of proinflammatory cytokines and chemokines in the lung. Furthermore, type I IFN administration to the lung potentiated inflammation in mice. We suggest that type I IFN production and IFNAR receptor signaling not only induce an antiviral state but also serve to amplify proinflammatory responses in the respiratory tract.

## MATERIALS AND METHODS

### Mice, virus stocks, TLR agonists, cytokines, and infection.

Six- to 10-week-old C57BL/6 (Harlan or Charles River, United Kingdom) or IFNAR1^−/−^ mice on a C56BL/6 background (obtained from C. Reis e Sousa, London Research Institute, London, United Kingdom) were maintained under pathogen-free conditions under UK Home Office guidelines.

Plaque-purified human RSV (originally strain A2 from the ATCC, United States) was grown in HEp-2 cells (20). Age- and sex-matched mice were lightly anesthetized and infected intranasally (i.n.) with 2 × 10^6^ focus-forming units (FFU) of RSV in 100 μl. For lung challenge with innate stimuli, CpG (1.25 μg/g bodyweight), poly(I·C) HMW (high molecular weight; 3.5 μg/g), and lipopolysaccharide (LPS; 500 or 50 ng/g) in 100 μl were administered i.n. (all from Invivogen). Recombinant IFN-α11 (Miltenyi Biotech) was administered i.n. at 500 ng/mouse.

### Cell collection and preparation.

Bronchoalveolar lavage (BAL) was carried out by flushing 3 times with 1 ml phosphate-buffered saline (PBS) containing 0.5 mM EDTA. For determination of cellular composition in the BAL fluid, cells were transferred onto a microscope slide (Thermo Scientific) using a cytospin centrifuge and stained with hematoxylin and eosin (H&E; Reagena).

### Chemokine and cytokine detection.

Chemokines and cytokines were quantified by a 20-plex Luminex kit according to the manufacturer's instructions (Life Technologies), and data were acquired with a Bio-plex 200 system (Bio-Rad laboratories, United Kingdom). The concentration of cytokines in each sample was determined according to the standard curve using the Bio-plex 6 software (Bio-Rad laboratories). IFN-λ2/3 (R&D), CXCL1 (R&D), or IFN-α (21) levels in the BAL fluid were measured by enzyme-linked immunosorbent assay (ELISA). Data were acquired on a SpectraMax Plus plate reader (Molecular Devices) and analyzed using SoftMax software (version 5.2).

### Gene expression.

Lungs were homogenized in TRIzol (Invitrogen) using a TissueLyser LT (Miltenyi Biotech). RNA extractions were performed with chloroform-isopropanol. Removal of DNA from TRIzol-extracted RNA was carried out using the DNA-free kit according to the manufacturer's manual (Ambion). RNA yields were determined by using a NanoDrop 1000 spectrophotometer. cDNA conversion was performed using a high-capacity RNA-to-cDNA kit according to the manufacturer's instructions (Applied Biosystems). To quantify mRNA levels in lung tissue, real-time quantitative PCR (RT-qPCR) was performed. For mRNA analysis, the following primers and probes were used: for *Tnfa*, forward primer, 5′-CATCTTCTCAAAATTCGAGTGACAA-3′, reverse primer, 5′-TGGGAGTAGACAAGGTACAACCC-3′, and probe, 5′-FAM-CACGTCGTAGCAAAC-TAMRA-3′; and for *Ifng*, forward primer, 5′-TCAAGTGGCATAGATGTGGAAGAA-3′, reverse primer, 5′-TGGCTCTGCAGGATTTTCATG-3′, and probe, 5′-FAM-TCACCATCCTTTTGCCAGTT-TAMRA-3′. Quantitative PCRs (qPCRs) for *Ifnl2/3*, *Ifnb*, *Rsad2* (Viperin), *Oas1a*, *Eif2ak2* (PKR), and L gene were performed using primers and probes previously described (20, 22–24). The assay was performed using the Quantitect Probe PCR master mix (Qiagen) and the 7500 Fast real-time PCR system (Applied Biosystems). For absolute quantification, the exact number of copies of the gene of interest was calculated using a plasmid DNA standard curve for each gene. Results were normalized using *Gapdh* (encoding glyceraldehyde-3-phosphate dehydrogenase) as a housekeeping gene (Applied Biosystems). For relative quantification, the expressions of *Ddx58* (RIG-I), *Cxcl10*, *Cxcl1*, *Il6*, *Il1b*, and *Mx1* (all from Applied Biosystems) relative to the housekeeping gene *Gapdh* were determined. The Δ*C_T_* (cycle threshold difference) between the target gene and *Gapdh* was calculated for each sample and expressed as 2^−Δ*Ct*^. Analyses were performed using 7500 Fast System SDS software (Applied Biosystems).

### Statistical analysis.

Results are presented as means ± standard errors of the means (SEM). The significance of results between the groups was analyzed by two-tailed, nonparametric, unpaired Mann-Whitney *t* test (Prism software; Graph-Pad Software Inc.) and is indicated in the figures as follows: *, *P* < 0.05; **, *P* < 0.01; ***, *P* < 0.001). *P* values of <0.05 were considered significant.

## RESULTS

### Type I, II, and III interferon production is abrogated in IFNAR1^−/−^ mice infected with RSV.

**FIG 1:**
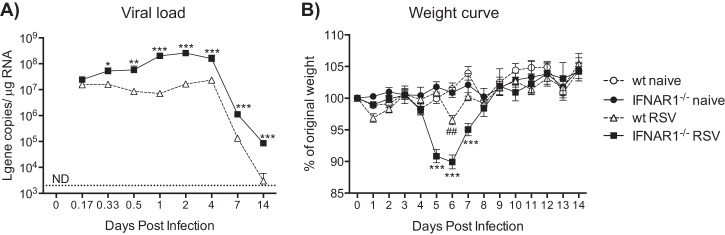
Viral load and weight loss after RSV infection. C57BL/6 wt and IFNAR1^−/−^ mice were infected intranasally with 2 × 10^6^ FFU of RSV. RNA was isolated from lung tissue, and after conversion into cDNA, copy numbers of RSV L gene RNA were determined using quantitative PCR (qPCR). (A) Levels of RSV L gene RNA in the lung tissue of wt and IFNAR1^−/−^ mice at different time points after RSV infection. Copy numbers were determined using a plasmid standard. Data shown are pooled data from 3 individual experiments with 3 to 5 mice per group in each experiment. The dotted line represents the detection limit. ND, not detectable. (B) Weights of infected and noninfected mice were monitored daily and plotted as a percentage of weight on the day of infection (day 0). The data shown are representative of at least 3 experiments with 3 to 5 mice per group in each experiment. Error bars indicate the SEM. ***, *P* ≤ 0.001; **, *P* ≤ 0.01; *, *P* ≤ 0.05 comparing RSV-infected wt with RSV-infected IFNAR1^−/−^ mice. ##, *P* ≤ 0.01 comparing RSV-infected wt with naive wt mice.

In order to investigate the role of IFNAR signaling in lung inflammation induced by infectious challenge, wild-type (wt; C57BL/6) and IFNAR1-deficient (IFNAR1^−/−^) mice were infected i.n. with RSV. We first assessed the effect of IFNAR deficiency on viral control. IFNAR1^−/−^ mice showed a significantly higher viral load in the lung, as measured by the copy number of viral L gene RNA, compared to wt mice from 8 h postinfection (p.i.) until day 14 p.i. (the latest time point studied; Fig. 1A). A delay in viral clearance was also apparent, as 11 of 12 IFNAR1^−/−^ mice had detectable L gene in the lungs at day 14 p.i. compared to only 1 of 12 mice in the wt group (Fig. 1A). The increase in lung viral load was accompanied by greater weight loss, a measure of infection severity (25): IFNAR1^−/−^ mice started to lose weight at day 5, lost significantly more weight than infected wt mice, and had a slower recovery (Fig. 1B).

**FIG 2:**
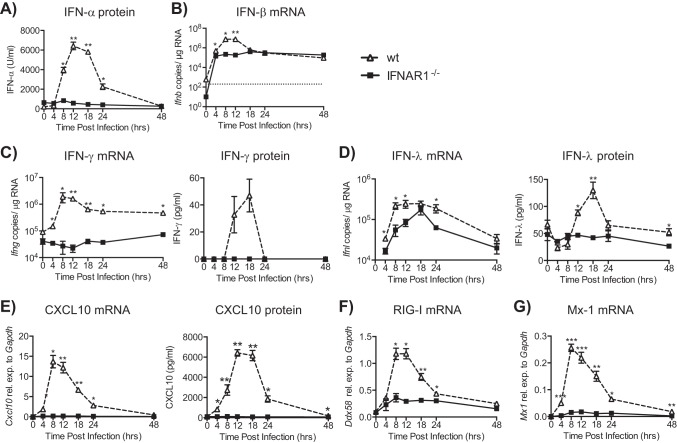
Interferon responses after RSV infection of IFNAR1^−/−^ and wt mice. C57BL/6 wt and IFNAR1^−/−^ mice were intranasally infected with 2 × 10^6^ FFU of RSV. (A) The level of IFN-α was determined in bronchoalveolar lavage (BAL) fluid by ELISA. (B) RNA was isolated from lungs, and gene expression levels of IFN-β were determined by qPCR. (C to E) IFN-γ (C), IFN-λ (D), and interferon-stimulated gene (ISG) CXCL10 (E) gene expression levels in lung tissue and protein production in BAL fluid were quantified. (F and G) RIG-I (*Ddx58*) (F) and Mx-1 (G) mRNA was determined by qPCR. Gene expression relative to GAPDH was calculated for CXCL10, RIG-I, and Mx-1. For IFN-β, IFN-λ, and IFN-γ, copy numbers were determined using a plasmid standard. The detection limit for all assays was 200 copies and is represented by the dotted line. Data shown are representative of at least 2 experiments with 4 or 5 mice per group. Error bars indicate the SEM. Significance when comparing RSV-infected wt with RSV-infected IFNAR1^−/−^ mice: **, *P* ≤ 0.01; *, *P* ≤ 0.05.

We then assessed the levels of IFNs in the lung early after infection by mRNA analysis and protein detection. IFN-α in the BAL fluid of wt mice was detected from 8 h p.i., with peak production at 12 to 18 h p.i. In contrast, no IFN-α was detectable in IFNAR1^−/−^ mice after RSV infection or in wt mice inoculated with UV-inactivated RSV (Fig. 2A and data not shown). Both wt and IFNAR1^−/−^ mice displayed similar levels of IFN-β mRNA at 4 h p.i., but this increased 100-fold at later time points in wt but not IFNAR1-deficient mice (Fig. 2B). This is consistent with the known IFNAR-dependent positive-feedback loop for type I IFN production (26). IFN-γ (mRNA or protein) was not detected in IFNAR1^−/−^ mice at any time point (Fig. 2C), in contrast to wt mice, which showed IFN-γ levels peaking at 12 and 18 h p.i. for mRNA and protein, respectively. Furthermore, although expression of IFN-λ mRNA was induced in IFNAR1^−/−^ mice, it was manifestly lower than in wt controls and did not result in detectable protein in BAL fluid (Fig. 2D). These data confirm and extend a previous study showing that expression of type I IFNs is reduced in RSV-infected IFNAR1^−/−^ mice (9).

We assessed whether the reduced levels of all IFNs in IFNAR1^−/−^ mice impacted the expression of selected interferon-stimulated genes (ISGs). CXCL10 could not be detected at either the mRNA or the protein level in lungs of IFNAR1^−/−^ mice but was induced in wt mice (Fig. 2E). *Ddx58* (RIG-I) mRNA levels increased above basal expression at 4 h p.i. in both IFNAR1^−/−^ and wt mice but continued to rise only in the wt mice until 12 h p.i. (Fig. 2F). Similar results were obtained with *Mx-1* (Mx-1; Fig. 2G), *Rsad2* (Viperin), *Oas1a* (OAS1), and *Eif2ak2* (PKR) gene expression (data not shown). In sum, our data suggest that the loss of type I IFN receptor signaling results in decreased expression of all IFN types early after RSV infection and prevents appropriate induction of ISGs. This is associated with a failure to control RSV replication and clear the virus rapidly, leading to increased pathology (weight loss).

### Proinflammatory cytokine responses are diminished in IFNAR1^−/−^ mice infected with RSV.

**FIG 3:**
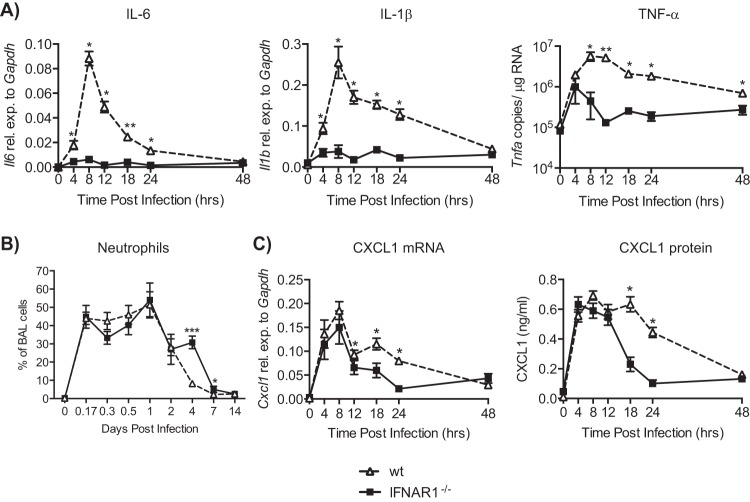
Diminished induction of proinflammatory cytokine mRNA in IFNAR1^−/−^ mice during RSV infection. C57BL/6 wt and IFNAR1^−/−^ mice were intranasally infected with 2 × 10^6^ FFU of RSV. (A) RNA was isolated from lungs, and gene expression levels of IL-6, IL-1-β, and TNF-α were determined by qPCR. (B) Percentage of neutrophils in the BAL fluid at indicated days postinfection. Data shown are pooled data from 3 individual experiments with 3 to 5 mice per group in each experiment. (C) CXCL1 (KC) gene expression levels in lung tissue and protein production in BAL fluid were quantified. Gene expression relative to GAPDH was calculated for IL-6, IL-1β, and CXCL1. For TNF-α, copy numbers were determined using a plasmid standard. Dotted lines represent the detection limit. Data shown are representative of at least 2 experiments with 4 or 5 mice per group, or for CXCL1 data shown are pooled data from 2 individual experiments with 3 to 5 mice per group in each experiment. Error bars indicate the SEM. Significance when comparing RSV-infected wt with RSV-infected IFNAR1^−/−^ mice: ***, *P* ≤ 0.001; **, *P* ≤ 0.01; *, *P* ≤ 0.05.

To further evaluate the inflammatory response early after RSV infection, we measured additional cytokines, including IL-6, IL-1β, and TNF-α. Very little, if any, mRNA encoding these cytokines could be detected in the lungs of RSV-infected IFNAR1^−/−^ mice (Fig. 3A). In contrast, such mRNAs were easily detectable in wt mice as early as 4 h p.i., with a peak of expression at 8 h p.i. (Fig. 3A). Early infiltration of neutrophils did not differ between wt and IFNAR1^−/−^ mice (Fig. 3B), so we also analyzed the expression of CXCL1 (KC), a known neutrophil attractant. CXCL1 mRNA and protein were detected at similar levels in both wt and IFNAR1^−/−^ mice at early time points (Fig. 3C). However, later during the infection CXCL1 levels were significantly higher in wt mice (Fig. 3C).

**FIG 4:**
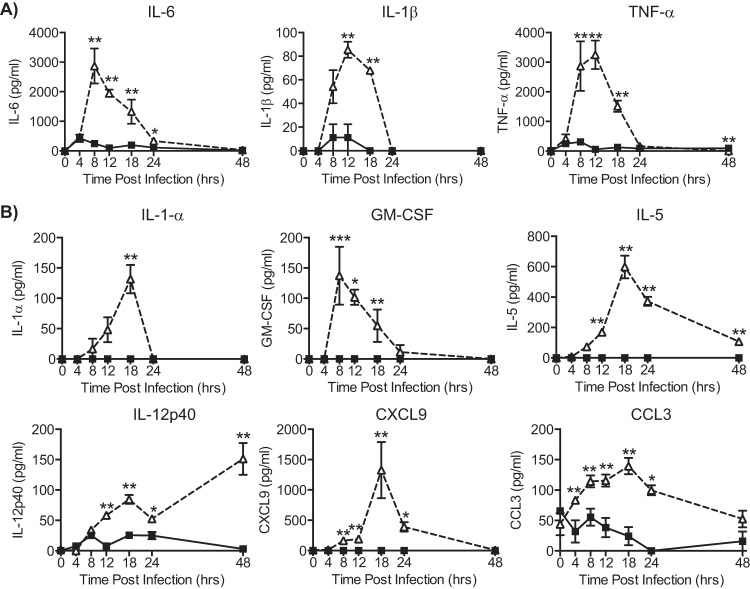
Reduced levels of proinflammatory cytokines in IFNAR1^−/−^ mice during RSV infection. C57BL/6 wt and IFNAR1^−/−^ mice were intranasally infected with 2 × 10^6^ FFU of RSV. At different time points postinfection protein levels of IL-6, IL-1β, TNF-α, IL-1α, GM-CSF, IL-5, IL-12p40, CXCL9, and CCL3 production were determined in BAL fluid by Luminex. Data shown are representative of at least 2 experiments with 4 or 5 mice per group. Error bars indicate the SEM. Significance when comparing RSV-infected wt with RSV-infected IFNAR1^−/−^ mice: **, *P* ≤ 0.01; *, *P* ≤ 0.05.

Levels of proinflammatory cytokines and chemokines were additionally measured in BAL fluid using a multiplex approach. IL-2, IL-4, IL-10, IL-13, and IL-17A were not detected in the airways of either wt or IFNAR1^−/−^ mice at any time point after infection (data not shown). For some cytokines (IL-6, IL-1β, TNF-α, and IL-12p40), there were measurable levels in IFNAR1^−/−^ mice at 4 to 8 h p.i., but this was not comparable to the levels in wt mice, which were 5 to 10 times greater (Fig. 4). For other cytokines and chemokines such as IL-1α, granulocyte-macrophage colony-stimulating factor (GM-CSF), IL-5, IL-12p40, CXCL9, and CCL3, there was no or very little induction in the airways of IFNAR1^−/−^ mice at any time point, while wt mice showed substantial levels peaking at 8 to 12 h p.i. (Fig. 4B). Overall, these results indicate that the lack of type I IFN receptor signaling results in a marked reduction in induction of proinflammatory mediators in lung and airways upon pulmonary viral infection.

### IFNAR1-deficient mice display reduced proinflammatory cytokine responses to lung challenge with TLR agonists.

**FIG 5:**
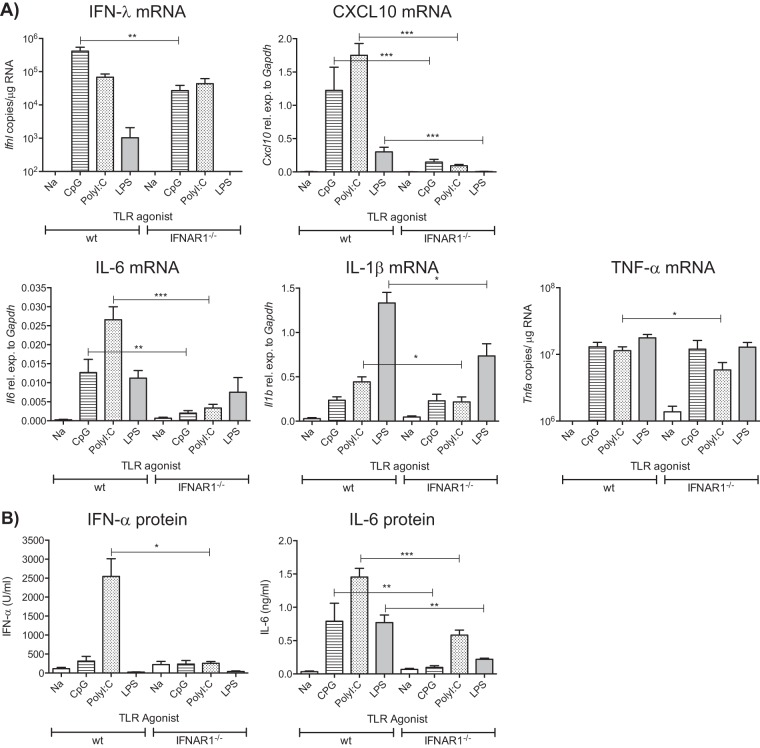
Induction of cytokines after intranasal challenge of IFNAR1^−/−^ and wt mice with innate stimuli. C57BL/6 wt and IFNAR1^−/−^ mice were intranasally challenged with TLR agonists [CpG, 1.25 μg/g body weight; poly(I·C), 3.5 μg/g body weight; LPS, 500 ng/g body weight]. Lungs and BAL fluid were collected 24 h postchallenge. (A) RNA was isolated from lungs, and gene expression levels of IFN-λ, CXCL10, IL-6, IL-1β, and TNF-α were determined by qPCR. Gene expression relative to GAPDH was calculated for IL-1β, IL-6, and CXCL10. For IFN-λ and TNF-α, copy numbers were determined using a plasmid standard. (B) IFN-α and IL-6 detected in the BAL fluid using ELISA. Data shown are pooled data from 2 individual experiments with 4 or 5 mice per group in each experiment. Error bars indicate the SEM. Significance when comparing RSV-infected wt with RSV-infected IFNAR1^−/−^ mice: ***, *P* ≤ 0.001; **, *P* ≤ 0.01; *, *P* ≤ 0.05.

To investigate whether similar results applied to noninfectious stimulation of the airways, we administered different innate immune stimuli intranasally (i.n.) to wt and IFNAR1^−/−^ mice. Mice were sacrificed 24 h following administration of CpG, poly(I·C), or LPS, and lung cytokine expression was measured using qPCR and ELISA. We observed significantly reduced mRNA levels of IFN-λ in response to the TLR9 agonist CpG and the TLR4 agonist LPS and of CXCL10 in response to all TLR agonists tested in IFNAR1^−/−^ mice (Fig. 5A). The expression of proinflammatory cytokines was also quantified. Decreased induction of IL-6, IL-1β, and TNF-α mRNA was observed with the TLR3, RIG-I, and MDA-5 agonist, poly(I·C), in IFNAR1^−/−^ mice. Moreover, a significant reduction in IL-6 mRNA was seen in IFNAR1^−/−^ mice treated with CpG compared to wt mice (Fig. 5A). Similarly, LPS-dependent induction of IL-1β mRNA was reduced in lungs of IFNAR1^−/−^ mice (Fig. 5A). A similar pattern was detected at the protein level: IFN-α was not induced after poly(I·C) treatment, and IL-6 was not induced by any of the TLR agonists in IFNAR1^−/−^ mice (Fig. 5B). Thus, the lack of type I IFN receptor decreases the lung inflammatory response provoked by innate stimulation of the airways.

### IFN-α potentiates proinflammatory cytokine induction.

**FIG 6:**
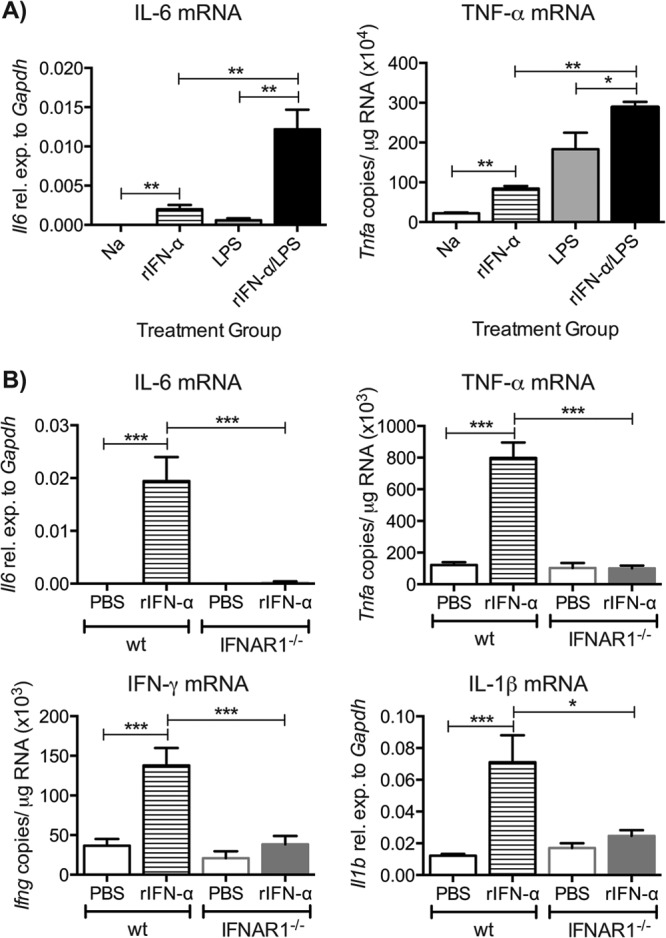
Recombinant IFN-α can amplify proinflammatory cytokine induction. (A) C57BL/6 wt mice were intranasally challenged with a suboptimal dose of LPS (50 ng/g body weight) with or without recombinant IFN-α11 (rIFN-α11) (500 ng/mouse), and lungs were collected after 24 h. (B) C57BL/6 wt and IFNAR1^−/−^ mice were intranasally challenged with rIFN-α (500 ng/mouse), and lungs were collected after 12 h. RNA was isolated from lungs, and gene expression levels of IL-6 and TNF-α (A) and IL-6, TNF-α, IFN-γ, and IL-1β (B) were determined by qPCR. Gene expression relative to GAPDH was calculated for IL-6 and IL-1β, and for TNF-α and IFN-γ copy numbers were determined using a plasmid standard. Data shown are pooled data from 2 individual experiments with 4 or 5 mice per group in each experiment. Error bars indicate the SEM. Significance when comparing RSV-infected wt with RSV-infected IFNAR1^−/−^ mice: ***, *P* ≤ 0.001; **, *P* ≤ 0.01; *, *P* ≤ 0.05.

The above results suggested that type I IFNs might potentiate proinflammatory responses in the lung. To explicitly test this hypothesis, one representative type I IFN, IFN-α11, was administered intranasally to mice concomitantly with innate immune challenge. The dose of IFN-α was similar to a dose previously used to inhibit RSV infection (8), and LPS was chosen as the stimulus because it does not induce high expression of type I IFNs in the lung (data not shown). Mice that received both IFN-α and a suboptimal dose of LPS showed increased lung expression of mRNAs encoding IL-6 and TNF-α (Fig. 6A) or IFN-γ (data not shown) 24 h postchallenge compared to mice that received only LPS or IFN-α. Interestingly, intranasal administration of IFN-α alone was sufficient to induce an increase in IL-6, TNF-α, IFN-γ, and IL-1β mRNA at 12 h (Fig. 6B) and IL-6 and TNF-α mRNA in the lung at 24 h (Fig. 6A). This was not observed in IFNAR1^−/−^ mice, indicating that the effect is dependent on signaling via IFNAR (Fig. 6) and not due, for example, to a contaminant. We conclude that type I IFNs markedly potentiate acute proinflammatory responses induced by innate immune stimulation of the airways.

## DISCUSSION

Type I IFNs are produced early after viral infection as a first line of host defense. They act on all cell types via the ubiquitously expressed IFNAR to induce increased expression of more than 300 different genes whose products eventually interfere with viral replication and viral spread, as well as lead to the initiation of immune responses (3, 10). Previous studies addressing the role of type I IFNs in the lung have focused mainly on adaptive immunity (8, 9, 19, 27, 28). Thus, the importance of type I IFNs signaling for the early innate immune response in the lung remains elusive. In this study, we uncover a general role for IFNAR signaling in amplifying acute lung inflammatory responses to innate stimuli. Further, we provide *in vivo* evidence for an important role of IFNAR in innate resistance to RSV lung infection and show that signaling through IFNAR is necessary for coordinating the inflammatory response to the virus. Our data suggest that type I IFNs are pivotal contributors to lung inflammation.

We anticipated that other IFNs might compensate for the lack of type I IFN signaling during RSV infection, as previously shown for influenza virus (9, 29–31), but were surprised to find that our data did not support this hypothesis; neither IFN-λ nor IFN-γ was upregulated in the lungs of IFNAR1^−/−^ mice during the early stages of RSV infection. Instead, expression of all IFNs and ISGs was decreased in RSV-infected IFNAR1^−/−^ mice compared to wt controls. This suggests that type I IFNs are involved in controlling the expression of IFN-α/β, IFN-γ, IFN-λ, and ISGs during RSV infection. For IFN-α/β, this is expected because type I IFN production relies on a positive-feedback loop through the type I IFN receptor (26). In addition, previous studies have shown that type I IFNs play a critical role in induction of IFN-γ gene expression through the activation of STAT4 (32, 33) or increased signaling through other cytokine receptors such as IFN-γ receptor by increased levels of STAT1 (19, 34). Furthermore, since the IFN responses were reduced in IFNAR1^−/−^ mice, this resulted in a diminished induction of ISGs as has previously been shown for TLR stimulation (35, 36) and for bone marrow-derived DCs (BMDCs) stimulated with RSV (37). Therefore, type I IFN production with subsequent IFNAR signaling is a key component of the entire IFN response early after RSV infection.

Surprisingly, our data indicate that type I IFN production and subsequent IFNAR signaling are also a key component of the entire inflammatory response. Indeed, we found that the induction of proinflammatory cytokines (e.g., IL-6, IL-1β, and TNF-α) was abrogated in the lungs of IFNAR1^−/−^ mice after RSV infection. A similar pattern was seen in BAL fluid for a broader array of proinflammatory cytokines and chemokines. Furthermore, IFNAR deficiency decreased the induction of proinflammatory cytokines in response to airway challenge with different innate immune stimuli, and IFN-α augmented the proinflammatory response to LPS stimulation. Also, IFN-α alone given intranasally drove a rapid and transient induction of proinflammatory cytokines in the lung. It has been shown that bone marrow-derived macrophages can produce CCL2 after stimulation with IFN-β (38) and that recombinant IFN-α (rIFN-α) potentiates serum TNF-α response to LPS administration (39). Furthermore, the dependence on IFNAR signaling for cytokine production has previously been suggested in studies where BMDCs from IFNAR1^−/−^ mice were found to produce less IL-12p70 after RSV exposure (37) or after treatment with select combinations of TLR agonists (36). However, it is possible that the effect of IFN-α in the lung is not to initiate *de novo* cytokine synthesis but to amplify that which has been initiated by other stimuli such as environmental endotoxins or airway commensals. Whichever the case, our data point to a hitherto unappreciated key role for IFNAR signaling in amplifying lung inflammation. This has been noted in systemic models of inflammation, where IFNAR1^−/−^ mice have been shown to be more resistant to LPS-induced septic shock and to lethal immunopathology induced by systemic Candida albicans infection (3, 40, 41).

There are several possible mechanisms that could explain our findings. First, proinflammatory cytokines are regulated mainly by the transcription factor NF-κB. Synergy between IFNAR signaling and NF-κB pathways has been suggested (36), and a recent report revealed multiple IFN-stimulated pathways that can activate NF-κB (42). However, NF-κB is activated at 0.5 to 1.5 h after RSV inoculation independently of viral replication (43). This could explain the early induction of some cytokines such as IFN-β, TNF-α, and CXCL1, which were detected at 4 h p.i. in IFNAR1^−/−^ mice. Another possibility is that the expression of molecules involved in virus recognition (e.g., RIG-I), signaling (e.g., MyD88), and cytokine production (e.g., MTOR) (44) is dependent on type I IFN receptor signaling. A third possibility is that the cellular source of the proinflammatory cytokines needs to be recruited into the lung and that this recruitment is dependent on IFNAR signaling. That source is unlikely to be neutrophils, as we observed comparable induction of CXCL1 and early infiltration of neutrophils into the airways of RSV-infected IFNAR1^−/−^ and wt mice.

Proinflammatory cytokines are known to cause discomfort and reduce appetite, leading to weight loss (10, 45). Oddly, we observed increased weight loss during RSV infection in IFNAR1^−/−^ mice, even though proinflammatory cytokines were not detected. The weight loss during RSV infection coincides with the infiltration of T cells (days 5 to 7 p.i.) and is reduced if T cells are depleted (46). However, during both RSV and Sendai virus infections, virus-specific T cells in IFNAR1^−/−^ mice are generated in numbers comparable to those seen in wt mice (references 19 and 47 and data not shown). Therefore, we suppose that the increased weight loss in the IFNAR1^−/−^ mice is not a manifestation of the immune response but, rather, a direct consequence of increased viral load and possible associated cytopathic damage to the lung structure and epithelial barrier.

The notion that weight loss can be a direct manifestation of RSV load has been previously suggested (25). Consistent with that notion, we found that IFNAR1^−/−^ mice were more permissive to RSV infection both as measured by weight loss and as measured by quantitating viral RNA in the lungs. This is in contrast to results from other groups who have found no effect of IFNAR in the resistance to RSV (19, 31) and might be explained by the use of distinct mouse strains (19), volumes and virus titers used for infections, virus purity (31), and/or methods for viral detection (qPCR versus plaque assay). In addition, differences in microbiota among mice from different animal facilities might be a contributing factor, as increasingly appreciated (48).

In summary, our study shows that lack of signaling through the IFNAR has a negative impact on the production of proinflammatory cytokines in the lung both after exposure to different innate stimuli and during RSV infection. This reveals a dual role for type I IFNs during respiratory infections, limiting viral replication while at the same time regulating the cytokine milieu. Furthermore, it suggests that an excessive induction of type I IFNs could potentially be detrimental for the host by driving excessive lung inflammation. The demonstration that type I IFNs can amplify lung inflammation when given exogenously may have applications in the design of vaccines or therapeutics for use at mucosal surfaces; conversely, local IFN blockade might be deployed as a strategy by which to limit lung inflammation.
